# Supplementary material for: Osteoporosis as the Female-Specific Risk Factor for Dynapenia in Elderly Patients with Type 2 Diabetes
Source: J Clin Med. 2024 Aug 6;13(16):4590. doi: 10.3390/jcm13164590 (PMC11354462; doi:10.3390/jcm13164590)
Supplement: Supplementary file 1 [file jcm-13-04590-s001.zip › JCM -3006238 - Supplementary Table S1.pdf]

Supplementary Table S1. Basic characteristics, medical prescription, grip strength and DXA scan parameters in the T2DM patients

	Females (n = 60)	Males (n = 43)	*p* value
Age (years)	67.5 ± 7.1	65.5 ± 7.1	0.164
Years of diabetes	13.1 ± 8.7	12.5 ± 8.9	0.719
BMI (kg/m^2)	24.9 ± 3.5	26.6 ± 4.2	0.082
Systolic BP (mmHg)	134 ± 17	135 ± 19	0.807
Diastolic BP (mmHg)	74 ± 9	77 ± 10	0.095
Fasting glucose (mg/dL)	140 ± 51	132 ± 29	0.896
HbA1c %	7.7 ± 1.4	7.4 ± 1.0	0.388
LDL cholesterol (mg/dL)	91 ± 27	86 ± 26	0.360
Triglyceride (mg/dL)	120 ± 68	118 ± 52	0.960
Creatinine (mg/dL)	1.1 ± 1.7	1.3 ± 1.0	**<0.001****
ALT (U/L)	21 ± 10	22 ± 12	0.804
Insulin % (n)	35 % (21)	27.9 % (12)	0.447
Metformin % (n)	73.3 % (44)	83.7 % (36)	0.212
SU or glinide % (n)	51.7 % (31)	62.8 % (27)	0.262
DPP4i % (n)	33.3 % (20)	27.9 % (12)	0.557
TZD % (n)	6.7 % (4)	14 % (6)	0.218
SGLT2i % (n)	26.7 % (16)	44.2 % (19)	0.064
GLP1RA % (n)	13.3 % (8)	11.6 % (5)	0.797
Grip strength (kg)	18.9 ± 5.6	33.2 ± 9.2	**<0.001****
**DXA scan parameters**			
Total lean mass (kg)	35.1 ± 4.3	51.0 ± 7.0	**<0.001****
Total BMC (kg)	1.95 ± 0.37	2.72 ± 0.46	**<0.001****
Total fat mass (kg)	20.8 ± 6.7	21.2 ± 9.4	0.831
Arms fat to lean ratio	0.63 ± 0.19	0.32 ± 0.16	**<0.001****
Legs fat to lean ratio	0.51 ± 0.19	0.31 ± 0.15	**<0.001****
Android/gynoid fat ratio	0.68 ± 0.16	0.84 ± 0.19	**<0.001****
Lumbar BMD (g/cm^2)	1.06 ± 0.21	1.19 ± 0.20	**0.001***
Left femur BMD (g/cm^2)	0.85 ± 0.14	0.96 ± 0.14	**<0.001****
Right femur BMD (g/cm^2)	0.86 ± 0.14	0.96 ± 0.13	**<0.001****

Continuous variables were analyzed using the Mann-Whitney U-test and are presented as mean values ± standard deviation; Categorical variables were analyzed using the Chi-square test and are presented as percentages (number). Abbreviations: BMI, body mass index; BP, blood pressure; HbA1c, glycated hemoglobin; LDL, low density lipoprotein; ALT, alanine aminotransferase; SU, sulfonylurea; DPP4i, dipeptidyl peptidase 4 inhibitor; TZD, thiazolidinedione; SGLT2i, sodium-glucose co-transporter 2 inhibitor; GLP1RA, glucagon-like peptide 1 receptor agonist; DXA, dual-energy x-ray absorptiometry; BMC, bone mineral content; BMD, bone mineral density. *$p<0.05$; **$p<0.001$
