# Supplementary material for: Osteoporosis as the Female-Specific Risk Factor for Dynapenia in Elderly Patients with Type 2 Diabetes
Source: J Clin Med. 2024 Aug 6;13(16):4590. doi: 10.3390/jcm13164590 (PMC11354462; doi:10.3390/jcm13164590)
Supplement: Supplementary file 1 [file jcm-13-04590-s001.zip › Medical Editing & Review Certificate.pdf]

GwoJang Tech Inc.
Medical Editing & Review Certificate

Title:

Osteoporosis as the female-specific risk factor for dynapenia in elderly patients with type 2 diabetes

This is to certify that the document listed above has been edited to the standards of the industry.

Sincerely,

GwoJang Tech Inc.

TEL： +886 2 23514696
FAX：+886 2 23514696

kc2351@ms24.hinet.net
